# A new prediction model for sustained ventricular tachycardia in arrhythmogenic cardiomyopathy

**DOI:** 10.3389/fcvm.2024.1477931

**Published:** 2024-12-16

**Authors:** Baowei Zhang, Xin Xie, Jinbo Yu, Yizhang Wu, Jian Zhou, Xiaorong Li, Bing Yang

**Affiliations:** ^1^ Department of Cardiology, Shanghai East Hospital, Tongji University School of Medicine, Shanghai, China; ^2^ Department of Cardiology, Ji'an Center People’s Hospital, Ji'an, China; ^3^ Department of Cardiology, The First Affiliated Hospital of Nanjing Medical University, Nanjing, China

**Keywords:** arrhythmogenic cardiomyopathy, sustained ventricular tachycardia, sudden cardiac death, prediction model, nomogram

## Abstract

**Background:**

Arrhythmogenic cardiomyopathy (ACM) is an inherited cardiomyopathy characterized by high risks of sustained ventricular tachycardia (sVT) and sudden cardiac death. Identifying patients with high risk of sVT is crucial for the management of ACM.

**Methods:**

A total of 147 ACM patients were retrospectively enrolled in the observational study and divided into training and validation groups. The least absolute shrinkage and selection operator (LASSO) regression model was employed to identify factors associated with sVT. Subsequently, a nomogram was constructed based on multivariable logistic regression analysis. The performance of the nomogram was evaluated using the area under the curve (AUC) of the receiver operating characteristic (ROC) curve and calibration curve. Decision curve analysis was conducted to assess the clinical utility of the nomogram.

**Results:**

Seven parameters were incorporated into the nomogram: age, male sex, syncope, heart failure, T wave inversion in precordial leads, left ventricular ejection fraction (LVEF), SDNN level. The AUC of the nomogram to predict the probability of sVT was 0.867 (95% CI, 0.797–0.938) in the training group and 0.815 (95% CI, 0.673–0.958) in the validation group. The calibration curve demonstrated a good consistency between the actual clinical results and the predicted outcomes. Decision curve analysis indicated that the nomogram had higher overall net benefits in predicting sVT.

**Conclusion:**

We have developed and internally validated a new prediction model for sVT in ACM. This model could serve as a valuable tool to accurately identify ACM patients with high risk of sVT.

## Introduction

Arrhythmogenic cardiomyopathy (ACM), an inherited cardiomyopathy characterized by high risks of ventricular arrhythmias and sudden cardiac death, is one of the leading causes of SCD in young people and athletes (1–3). Sustained ventricular tachycardia (sVT) constitutes a major contributor to SCD in ACM patients. Although frequency catheter ablation (RFCA) and anti-arrhythmic drugs have effectively reduced sVT incidence, the implanted cardioverter defibrillator (ICD) remains the most efficacious therapy for preventing SCD in ACM patients (4). However, ACM often presents in adolescents or at young age, and ICD treatment is invasive, associated with various complications, and imposes a dual burden of physiological and psychological stress on patients. Therefore, patients should be thoroughly assessed before undergoing ICD treatment (5). ACM patients with a history of sVT or resuscitated SCD can benefit from secondary prevention with ICD. Nevertheless, for ACM patients without documented sVT, the primary goal in the management of ACM patients is to identify those at high risk of sVT and treated with prophylactic ICD implantation (6).

Previous studies have identified several risk factors for predicting sVT in ACM patients, including gender, proband status, cardiac syncope, degree of exercise restriction, premature ventricular complex (PVC) burden, non-sustained VT, genetic testing, and extend of myocardial involvement (7, 8). Two studies have constructed prediction models for sudden cardiac death and sustained ventricular tachycardia in ACM patients using these risk factors (9, 10). These prediction models could effectively predict the occurrence of sVT and sudden cardiac death, but their predictive value was limited in ACM patients without identified pathogenic gene variants and in those with left ventricular involvement (11–13). These two prediction models considered demographic factors, symptoms, PVCs burden, and extend of myocardial involvement, but they overlooked the pathophysiological mechanisms underlying the occurrence of sVT in ACM patients. Increased cardiac sympathetic nerve activity plays an important role in the occurrence of sVT in ACM patients (14). Our preliminary study found that SDNN, an indicator of cardiac sympathetic nerve activity, could effectively predict the occurrence of sVT in ACM patients (15). The predictive value of SDNN is based on the role of hyperactivity of cardiac sympathetic nerve system in the occurrence of sVT, a mechanism distinct from other traditional risk factors. Therefore, we hypothesized that incorporating SDNN in the prediction model could provide additional value in distinguishing ACM patients with high risk of sVT.

In the present study, a total of 147 ACM patients were retrospectively enrolled and divided into training and validation groups. A prediction model was built on the basis of multivariable logistic regression analysis, and performed well in identifying ACM patients with high risk of sVT in both the training and validation groups.

## Methods

### Patients

A total of 212 patients diagnosed with ACM were retrospectively enrolled from the First Affiliated Hospital of Nanjing Medical University and Shanghai East Hospital between January 2006 and October 2024. This study was conducted in accordance with the principles outlined in the Declaration of Helsinki (revised in 2013) and received approval from the institutional ethics committee board of the First Affiliated Hospital of Nanjing Medical University (Approval No. 2011-SR-014). Given the retrospective nature of the study, individual consent was waived. Diagnosis of ACM was based on the updated diagnostic criteria (6, 16). Patients with incomplete data, a history of heart transplantation (having received a normal heart), or other comorbidities associated with a high risk of ventricular arrhythmias (e.g., LVEF <35%) were excluded from the study.

### Data collection

All enrolled ACM patients underwent retrospective evaluation of elementary clinical characteristics, as well as electrocardiographic and echocardiographic features. Elementary clinical data encompassed age, sex, age at diagnosis, prior syncope episodes, family history, comorbidities, and both pharmacological and non-pharmacological therapies. Surface electrocardiography (ECG), 24-h Holter monitoring, and echocardiography were meticulously reviewed by two independent physicians to identify potential predictors for risk stratification in ACM. The standard deviation of all normal-normal (NN) intervals (SDNN) was computed as the dispersion of all NN intervals over a 24-h period of Holter monitoring (17). Furthermore, all patients were meticulously assessed by two independent physicians to detect evidence of sVT. sVT was defined as follows: (1) recorded spontaneous persistent ventricular tachycardia (lasting for ≥30 s at ≥100 beats per minute, or with unstable hemodynamics requiring cardioversion), (2) ventricular fibrillation/flutter, or (3) appropriate ICD intervention in patients with such devices.

### Statistical analysis

For continuous variables, the Kolmogorov-Smirnov test was employed to assess the distribution of the data. Variables exhibiting a normal distribution are expressed as mean ± standard deviation (SD), with differences between groups investigated using the Student's *t*-test. Variables not conforming to a normal distribution are presented with interquartile range and median, and differences were examined using the Mann-Whitney test. Categorical data were summarized using percentages, and differences between groups were evaluated using the χ^2^ or Fisher's exact test, as appropriate.

The initial population was randomized into training and validation groups at a ratio of 7:3, which were subsequently utilized for model development and validation, respectively. The least absolute shrinkage and selection operator (LASSO) logistic regression model was employed to identify factors with significant predictive value for the occurrence of sVT from the data in the training group. Factors exhibiting nonzero coefficients in the LASSO regression model were retained. Subsequently, a multivariable logistic regression analysis, incorporating factors selected from the LASSO regression as well as other clinically significant variables, was conducted to construct a predictive model. To maximize the inclusion of effective predictive factors, variables with a two-sided *p*-value ≤0.1 were incorporated into the prediction model, while factors of evident clinical significance were directly included. To provide quantitative predictions of sVT probability for individual patients, a nomogram was constructed based on the multivariable logistic regression analysis conducted in the training group.

To evaluate the discriminatory capability of the nomogram, the area under the curve (AUC) of the receiver operating characteristic (ROC) curve and calibration curve analyses were conducted in the training group. These methods were also applied to the validation group to further validate the performance of the nomogram. Additionally, decision curve analyses (DCA) were performed to assess the clinical utility of the nomogram by quantifying the net benefits in both groups. All statistical analyses were conducted using R software (Version 4.2.0; https://www.r-project.org).

## Results

### Baseline characteristics

After excluding 65 patients for various reasons, a total of 147 ACM patients were ultimately enrolled in this study (Supplementary Figure S1). Among them, 60.5% of patients (89 individuals) had a history of sVT. Patients with and without sVT exhibited similar characteristics in terms of age, history duration, age at diagnosis, family history of ACM, comorbidities, and pharmacological therapies. However, patients with sVT demonstrated a higher proportion of males (87.6% vs. 56.9%, *p* < 0.001), smokers (29.2% vs. 6.9%, *p* = 0.002), alcohol consumers (15.7% vs. 3.4%, *p* = 0.04), users of class III anti-arrhythmic drugs (49.4% vs. 19.0%, *p* < 0.001), recipients of ICD devices (34.8% vs. 10.3%, *p* = 0.002), individuals who underwent RFCA (34.8% vs. 12.1%, *p* = 0.004), those with T wave inversion in anterior leads (73.0% vs. 48.3%, *p* = 0.004), right ventricular (RV) enlargement (68.5% vs. 32.8%, *p* < 0.001), and lower levels of SDNN [113.0 (89.0, 135.0) vs. 134.5 (113.0, 150.5) ms, *p* < 0.001], compared with patients without sVT (Table 1).

**Table 1:** Baseline characteristics of patients enrolled.

Variables	sVT group (*n* = 89, 60.5%)	Non-sVT group (*n* = 58, 39.5%)	*P*-value
Age (years)	45.9 ± 15.3	45.0 ± 15.8	0.72
Male (*n*, %)	78 (87.6)	33 (56.9)	<0.001
History (years)	3.0 (1.0, 10.0)	4.0 (1.0, 8.5)	0.91
Age of diagnosis (years)	42.0 (28.5, 48.0)	38.0 (28.5, 51.0)	0.93
Syncope (*n*, %)	39 (43.8)	21 (36.2)	0.46
ICD (*n*, %)	31 (34.8)	6 (10.3)	0.002
RFCA (*n*, %)	31 (34.8)	7 (12.1)	0.004
Family history (*n*, %)	8 (9.0)	3 (5.2)	0.59
Comorbidities			
Hypertension (*n*, %)	10 (11.2)	10 (17.2)	0.43
Diabetes (*n*, %)	0 (0)	3 (5.2)	0.12
Coronary artery disease (*n*, %)	2 (2.2)	0 (0)	0.67
Heart failure (*n*, %)	7 (7.9)	9 (15.5)	0.24
Smoking (*n*, %)	26 (29.2)	4 (6.9)	0.002
Alcohol (*n*, %)	14 (15.7)	2 (3.4)	0.04
Anti-arrhythmic drugs			
Class I (*n*, %)	15 (16.9)	7 (12.1)	0.58
Class II (*n*, %)	43 (48.3)	18 (31.0)	0.06
Class III (*n*, %)	44 (49.4)	11 (19.0)	<0.001
Class IV (*n*, %)	3 (3.4)	0 (0)	0.41
Characteristics on electrocardiography			
RBBB (*n*, %)	33 (37.1)	18 (31.0)	0.57
TWI-A (*n*, %)	65 (73.0)	28 (48.3)	0.004
TWI-I (*n*, %)	38 (42.7)	19 (32.8)	0.30
NSVT (*n*, %)	39 (43.8)	25 (43.1)	1.00
SDNN (ms)	113.0 (89.0, 135.0)	134.5 (113.0, 150.5)	<0.001
Characteristics on echocardiography			
RV enlargement (*n*, %)	61 (68.5)	19 (32.8)	<0.001
RVOT dyskinesia (*n*, %)	40 (44.9)	31 (53.4)	0.40
RV free wall dyskinesia (*n*, %)	63 (70.8)	34 (58.6)	0.18
PAH (*n*, %)	32 (36.0)	13 (22.4)	0.12
Tricuspid regurgitation (*n*, %)	63 (70.8)	32 (55.2)	0.08
Mitral regurgitation (*n*, %)	26 (29.2)	19 (32.8)	0.79
LVDD (mm)	47.0 (41.5, 50.0)	48.0 (45.0, 51.0)	0.09
LVEF (%)	61.0 (52.5, 65.8)	62.9 (55.0, 65.8)	0.59

sVT, sustained ventricular tachycardia; ICD, implantable cardiac defibrillator; RFCA, radiofrequency catheter ablation; RBBB, right bundle branch block; TWI-A, T wave inversion on anterior leads; TWI-I, T wave inversion on inferior leads; NSVT, non-sustained ventricular tachycardia; SDNN, Standard deviation of NN intervals; RV, right ventricle; PAH, pulmonary artery hypertension; LVDD, left ventricular end diastolic dimension; LVEF, left ventricular ejection fraction.

All 147 ACM patients were subsequently randomly divided into the training group (*n* = 104, 70.7%), with the remaining patients allocated to the validation group (*n* = 43, 29.3%). The incidence of sVT was found to be similar between the training and validation groups (56.7% vs. 69.8%, *p* = 0.20). Moreover, demographic characteristics, comorbidities, pharmacological therapies, electrocardiographic, and echocardiographic features were well-balanced between the training and validation groups (Supplementary Table S1).

### Feature selection and nomogram construction

**Figure 1:**
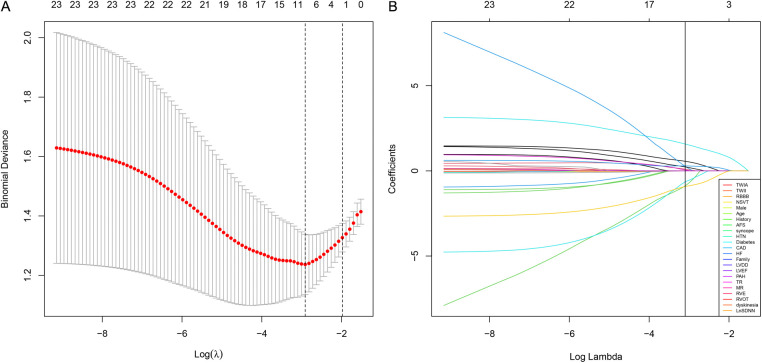
The least absolute shrinkage and selection operator (LASSO) regression model was employed to identify factors associated with sVT in ACM patients. **(A)** The optimal *λ* selection in the LASSO model was determined using 10-fold cross-validation, guided by the minimum criteria (the right dotted vertical line) and the 1-SE criteria (the left dotted vertical line). **(B)** The LASSO coefficient profiles for all 23 features revealed that eleven features with nonzero coefficients were selected at the optimal *λ*, as indicated by the vertical line.

In the training group, patients with sVT exhibited a higher proportion of males (93.2% vs. 55.6%, *p* < 0.001), smokers (32.2% vs. 4.4%, *p* = 0.001), alcohol consumers (18.6% vs. 0, *p* = 0.006), recipients of ICD devices (32.2% vs. 11.1%, *p* = 0.02), individuals who underwent RFCA (39.0% vs. 8.9%, *p* = 0.001), users of class III anti-arrhythmic drugs (AAD) therapy (54.2% vs. 24.4%, *p* = 0.004), those with T wave inversion in anterior leads (74.6% vs. 51.1%, *p* = 0.02), and RV enlargement (71.2% vs. 37.8%, *p* = 0.001), but exhibited lower levels of SDNN [114.0 (88.0, 136.0) vs. 132.0 (115.0, 151.0) ms, *p* = 0.003], consistent with findings observed in the entire cohort (Supplementary Table S2). The LASSO logistic regression model identified 11 features as potential predictors of sVT in the training group (Figure 1). Subsequently, a multivariable logistic regression analysis based on the factors selected in the LASSO regression was conducted to construct the prediction model. Additionally, for the comprehensive predictive value of sVT in ACM patients, syncope and left ventricular ejection fraction (LVEF) were also included. Ultimately, age, syncope, LVEF, male sex, heart failure, T wave inversion in anterior leads, and SDNN were incorporated into the prediction model (Table 2).

**Table 2:** Predictors for sVT in patients with ACM.

Variable	Prediction model		
	*β*	Odds ratio (95% CI)	*P*-value
TWI-A	1.38	3.97 (1.148–15.647)	0.04
LnSDNN	−2.19	0.11 (0.011–0.813)	0.04
Male	2.81	16.62 (3.613–108.370)	<0.001
Heart failure	−4.43	0.01 (0.001–0.155)	0.002
LVEF	−0.11	0.90 (0.818–0.978)	0.02
Syncope	1.01	2.73 (0.698–11.527)	0.15
Age	0.02	1.02 (0.970–1.071)	0.44

sVT, sustained ventricular tachycardia; ACM, arrhythmogenic cardiomyopathy; CI, confidence interval; TWI-A, T wave inversion on anterior leads; TWI-I, T wave inversion on inferior leads; NSVT, non-sustained ventricular tachycardia; LVDD, left ventricular end diastolic dimension; LVEF, left ventricular ejection fraction; RV, right ventricle; PAH, pulmonary artery hypertension; NSVT, non-sustained ventricular tachycardia; SDNN, Standard deviation of NN intervals; NA, not available.

**Figure 2:**
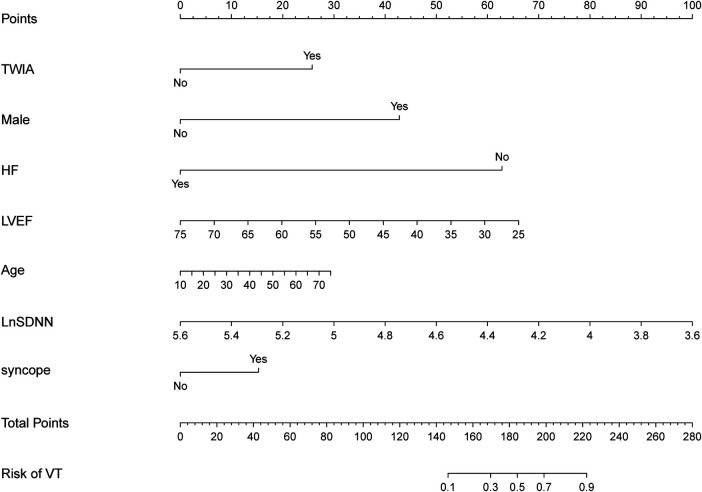
The nomogram was constructed in the training group for identifying ACM patients at high risk of sVT. Each factor in the nomogram was assigned an individual score based on its value, and the scores of all factors were summarized to a total score, which was used to estimate the risk of sVT for a given patient.

Based on the predictors identified by the LASSO logistic regression model in the training group, a nomogram was constructed in the training group for identifying ACM patients at high risk of sustained ventricular tachycardia (sVT). The nomogram included five significant predictive features and two canonical predictors (Figure 2). Each factor in the nomogram was assigned an individual score based on its value, and a total score was calculated by summing the scores of all factors. The final score derived from the nomogram could be utilized to estimate the risk of sVT for a given patient (Figure 2).

### Performance of the nomogram

**Figure 3:**
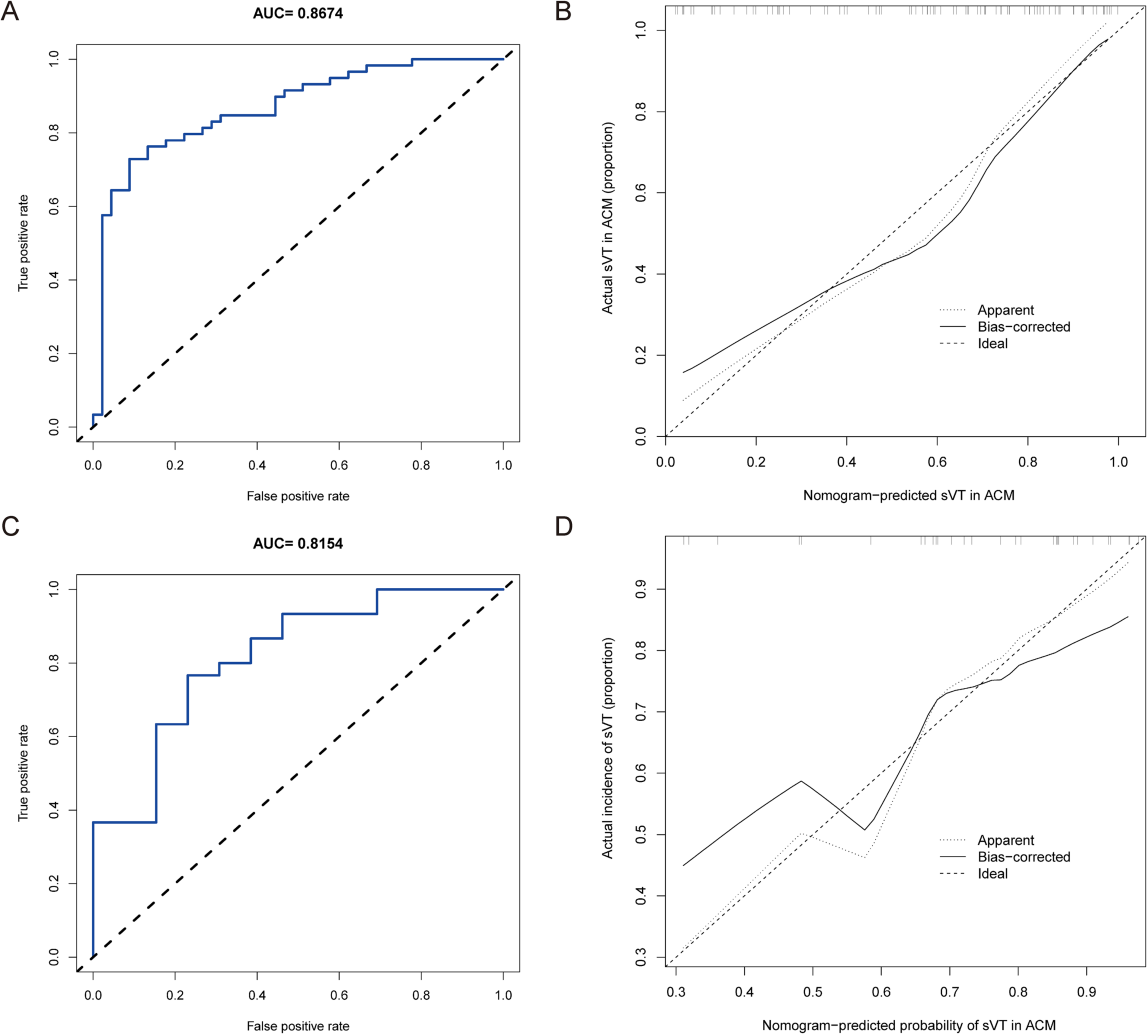
The nomogram was validated in both the training and validation groups. **(A)** The ROC curve was utilized to evaluate the performance of the nomogram in the training group. The AUC of the nomogram for predicting the occurrence of sVT in ACM patients was 0.867 (95% CI, 0.797–0.938). **(B)** The calibration curve in the training group demonstrated that the predictive nomogram provided excellent estimations of actual probabilities of sVT. **(C)** The ROC curve was used to evaluate the performance of the nomogram in the validation group. The AUC of the nomogram for predicting the occurrence of sVT in ACM patients was 0.815 (95% CI, 0.673–0.958). **(D)** The calibration curve in the validation group validated that the predictive nomogram provided excellent estimations of actual probabilities of sVT.

We firstly validated the predictive value of the nomogram for identifying ACM patients at high risk of sVT in the training group by means of the ROC curve. The AUC of the nomogram for predicting the occurrence of sVT in ACM patients was 0.867 (95% CI, 0.797–0.938) (Figure 3A). The calibration curve demonstrated that the predictive nomogram for the occurrence of sVT provided excellent estimations of actual probabilities (Figure 3B).

Subsequently, the performance of the prediction model was validated in the validation group. Among the 43 patients allocated to the validation group, 30 had a history of sVT (69.8%). Patients with sVT in the validation exhibited similar proportions of males, smokers, recipients of ICD devices, individuals who underwent RFCA, and those with T-wave inversion in anterior leads, which was different from patients in the training group (Supplementary Table S3). However, the AUC of the prediction model to forecast the incidence of sVT was 0.815 (95% CI, 0.673–0.958) in the validation group (Figure 3C), indicating that the prediction model demonstrated excellent performance in identifying ACM patients at high risk of sVT. Furthermore, the calibration curve also illustrated good consistency between the actual incidence of sVT and the predicted probability in the validation group (Figure 3D).

### Clinical utility

**Figure 4:**
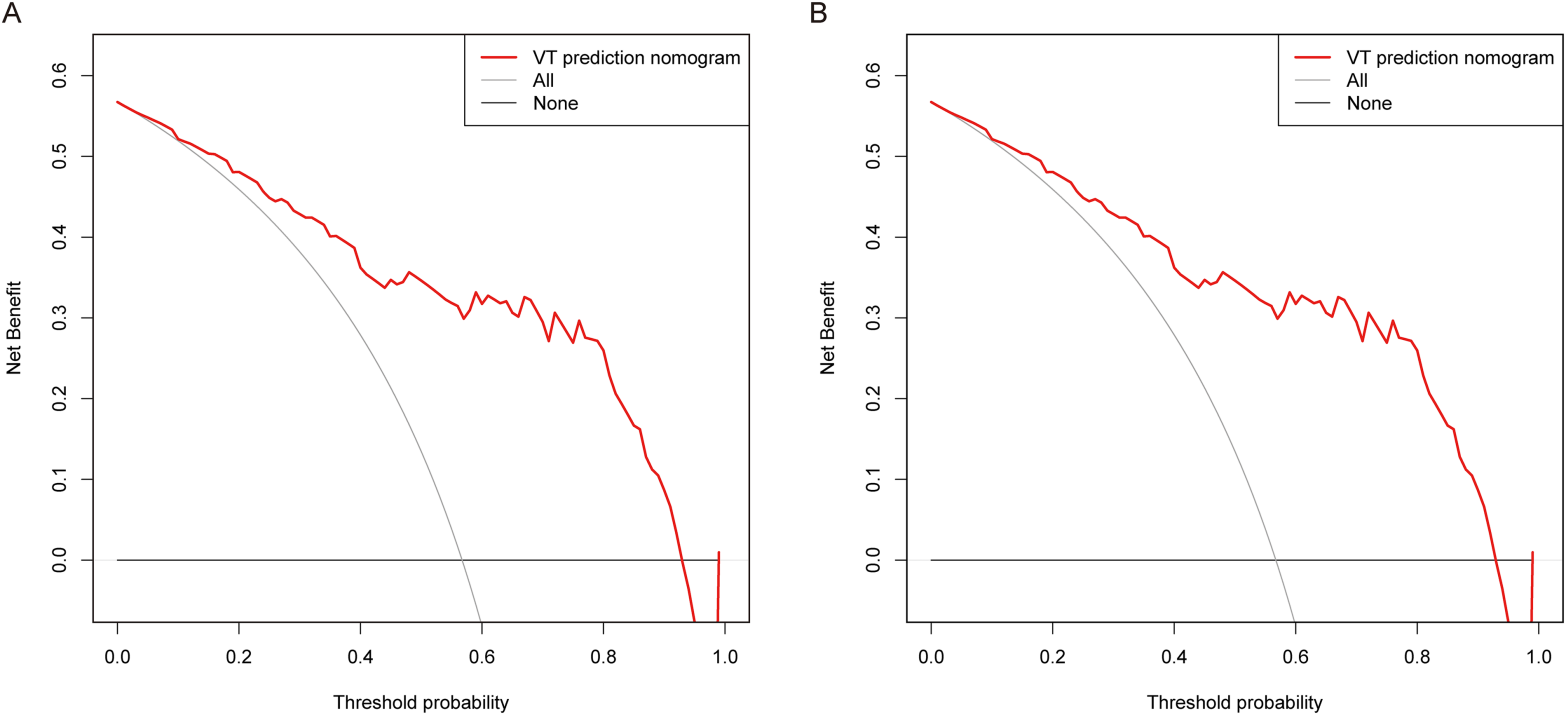
DCA was performed for the nomogram in both the training group **(A)** and the validation group **(B)** the red line indicated the predictive nomogram. The gray line represented the assumption that sVT occurred in all ACM patients, while the black line represented the assumption that no patient had sVT.

To explore the clinical implications of our predictive model, decision curve analyses were conducted in both the training group and the validation group. As depicted in Figure 4, the results of DCA illustrated that the predictive model provided excellent overall net benefits in predicting the incidence of sVT in ACM patients.

## Discussion

In this study, we developed and internally validated a prediction model for identifying ACM patients at high risk of sVT. All features incorporated into the prediction model consisted of elementary clinical information readily available in clinical practice, thus rendering the prediction model suitable for routine use in clinical settings. Predicted risks were consistent with the actual risks of sVT in both the training group [AUC 0.867 (95% CI, 0.797–0.938)] and the validation group [AUC 0.815 (95% CI, 0.673–0.958)]. Furthermore, decision curve analysis also demonstrated that the model was associated with a high net benefit in predicting the incidence of sVT in ACM patients.

High risk of sVT is the prominent clinical feature in patients with ACM. Previous studies have shown that patients with ACM exhibit annual sVT rates ranging from 2% to 10%, which are notably higher compared to patients with other non-ischemic cardiomyopathies (18–20). Substitution of the ventricular myocardium with fibrous or fibrofatty tissue represents a distinctive pathological feature in patients with ACM (21). The infiltration of fibrous or fibrofatty tissue disrupts the conduction velocity and direction of cardiac electrical activity, predisposing individuals to the formation of reentrant circuits and thereby increasing the risk of sVT (22). Premature ventricular beats and sVT exhibiting a left bundle branch block (LBBB) morphology with a non-inferior axis pattern in ACM patients indicate the “triangle of dysplasia” as the origin of ventricular arrhythmias, thereby supporting cardiac fibrosis as an important mechanism of sVT in ACM (23). However, clinical studies have shown that VAs could manifest before identifiable cardiac structural changes occur, suggesting the involvement of other mechanisms contributing to ventricular arrhythmias in ACM. Subsequent studies identified impairment of action potential upstroke velocity and propagation of action potential in cardiomyocytes from ACM mice, attributed to the dislocation of connexin 43 (Cx43) and dysfunction of sodium channel Nav1.5 (24–26). Furthermore, dysregulation of calcium (Ca^2+^) handling has been observed in cardiomyocytes from ACM mice (27). This disruption of Ca^2+^ homeostasis predisposes to sarcoplasmic reticulum spontaneous Ca^2+^ release and early afterdepolarizations, ultimately leading to increased cardiomyocyte excitability and the onset of ventricular arrhythmogenesis (28). Consequently, a combination of cardiac fibrosis, dislocation of Cx43 and Nav1.5, and disturbance of Ca^2+^ homeostasis is postulated to contribute to arrhythmogenesis in ACM.

In addition, the abnormal activity of sympathetic nerve system contributes to the ventricular arrhythmogenesis in ACM (29). Our clinical studies, along with others, have identified the overactivation of the cardiac sympathetic system in patients with ACM (14, 15, 30). Stimulation of myocardial β1 adrenergic receptors can increase cellular Ca^2+^ loading through the cyclic adenosine monophosphate (cAMP)–protein kinase A (PKA) pathway, resulting in an augmentation in the L-type calcium current, as well as through SERCA2-mediated reduction of Ca^2+^ re-uptake. The resultant elevation in diastolic Ca^2+^ levels within the cardiomyocyte nay heighten the likelihood of spontaneous sarcoplasmic reticulum calcium release, subsequently leading to the induction of afterdepolarization and extra stimulus, which could serve as triggers for inducing sVT (31). Additionally, the sprouting and remodeling of sympathetic nerve fibers secondary to sympathetic nerve fiber necrosis result in the heterogeneous reinnervation of cardiomyocytes, potentially promoting the dispersion of action potential duration and providing a suitable substrate for the maintenance of sVT (32). Therefore, the overactivity of the cardiac sympathetic nervous system not only provides triggers but also a suitable substrate for sVT in ACM patients.

Sustained ventricular tachycardia in ACM patients is associated with abnormal electroactivity of cardiomyocytes, cardiac fibrosis, and overactivation of the cardiac sympathetic system. Therefore, clinical parameters associated with these factors may serve as predictors of sVT in ACM patients. Previous studies have identified several predictors of sVT in ACM patients, including recent syncope, younger age at presentation, male sex, decreased left ventricular ejection fraction (LVEF), non-sustained ventricular tachycardia (NSVT), PVC burden, RV dysfunction, and leads with T-wave inversion (33–36). Syncope is a common initial symptom of ACM and often indicates that the patient has experienced hemodynamically unstable sVT, although non-cardiogenic causes of syncope should also be considered in some cases (37). Therefore, recent syncope is regarded as a significant indicator for identifying high-risk patients with sVT in ACM. NSVT and PVC burden may reflect the abnormal electroactivity of cardiomyocytes. Decreased LVEF and RV dysfunction may indicate more severe cardiac fibrosis and scar formation in both ventricles (38). Additionally, the 12-lead ECG plays a crucial role in the diagnosis of ACM, localization of ventricular arrhythmias, and identification of high-risk patients for sVT (39). T-wave inversion is not only one of the ECG criteria for diagnosing ACM, but it is also an independent factor in predicting the occurrence of sVT in patients with ACM (20, 40). This study also identified that T-wave inversion in the anterior leads was independently associated with sVT in ACM patients. A recent study integrated theses predictors and developed a prediction model for sVT in ACM patients (10). However, subsequent study showed that this prediction model had good performance in distinguishing patients with and without sVT in ACM patients with pathogenic mutant *PKP2* (C-index 0.77), but had limited value in predicting the risk of sVT in ACM patients with unknown pathogenic gene mutations (9). In addition, this predictive model only incorporated parameters related to the abnormal electroactivity of cardiomyocytes and cardiac fibrosis. We hypothesized that incorporation parameters associated with the overactivity of the cardiac sympathetic system might enhance the value of the prediction model.

SDNN, a parameter of heart rate variability, is regarded as an indicator of cardiac sympathetic system activity. Our previous study revealed that ACM patients exhibited decreased SDNN levels, and SDNN level emerged as an independent predictor of sVT in ACM patients (15). The present study also demonstrated that the SDNN levels in the sVT group were significantly lower than those in the control group. Further analysis using LASSO regression and logistic regression indicated that SDNN levels were independently associated with the occurrence of sVT in patients with ACM. The prediction model incorporating SDNN and these canonical parameters effectively identifies high-risk patients for sVT in individuals with ACM. Furthermore, calibration plots exhibited good agreement between predicted and observed sVT in ACM patients. Therefore, the predictive model in this study may more effectively identify high-risk patients for sVT among individuals with ACM.

The significant clinical benefit of our prediction model lies in its precise quantification of arrhythmic risk tailored to individual ACM patients, offering prognostic insights crucial for guiding clinical decisions regarding prophylactic ICD implantation. Additionally, all parameters included in the prediction model are readily accessible clinical variables, rendering the model user-friendly. It is important to acknowledge that all these parameters in the prediction model might change as ACM progresses. Thus, ACM patients should undergo periodic re-stratification as recommended in a recent expert consensus document.

## Limitations

One potential limitation is that all patients in this study were from tertiary medical centers in China and had relatively high incidences of sVT within this cohort (60.5%). The bias in patient selection could potentially impact the overall accuracy of this prediction model when applied to other ACM patients. Secondly, the relatively small sample size of patients used to develop a new prediction model might be associated with selection bias, reduce the statistical power, increase the risk of type II errors, and affect the reliability of the conclusions drawn. Although we have tried to minimize the impact of a small sample size by adding a validation cohort, caution is still required when interpreting and applying the results of our study. Additionally, due to its retrospective design, our study possesses certain drawbacks compared to a prospective study. Moreover, genetics play important roles in the diagnosis of ACM and sVT risk evaluation in ACM (41, 42). However, only a small portion of patients in this study underwent genetic testing, so genetics were not included in the construction of the prediction model. Finally, some *p*-values in this study were close to the significance threshold, raising the potential for type I error. Therefore, further large prospective cohort studies are needed to assess the accuracy and clinical utility of the prediction model in risk stratification for sVT in ACM patients.

In summary, we developed and validated a new prediction model to generate individualized estimates of the risk of incident sVT in ACM patients. Utilizing easily accessible clinical parameters, this model has the potential to accurately identify high-risk patients with sVT, thereby offering significant value for guiding prophylactic ICD implantation in ACM patients.

## Data Availability

The raw data supporting the conclusions of this article will be made available by the authors, without undue reservation.
