# Supplementary material for: A new prediction model for sustained ventricular tachycardia in arrhythmogenic cardiomyopathy
Source: Front Cardiovasc Med. 2024 Dec 16;11:1477931. doi: 10.3389/fcvm.2024.1477931 (PMC11683097; doi:10.3389/fcvm.2024.1477931)

Supplementary Material

A new prediction model for sustained ventricular tachycardia in arrhythmogenic cardiomyopathy

Supplementary table 1

Comparison of baseline characteristics of patients between model group and validation group

Variables	Model group (n = 104, 70.7%)	Validation group （n = 43, 29.3%）	P value
Age (years)	45.5 ± 15.0	45.8 ± 16.9	0.91
Male (n, %)	80 (76.9)	31 (72.1)	0.68
sVT (n, %)	59 (56.7)	30 (69.8)	0.20
History (years)	4.0 (1.0, 10.0)	3.0 (1.0, 10.0)	0.67
Age of diagnosis (years)	41.0 (29.3, 50.0)	43.0 (28.0, 48.0)	0.86
Syncope (n, %)	44 (42.3)	16 (37.2)	0.70
ICD (n, %)	24 (23.1)	13 (30.2)	0.48
RFCA (n, %)	27 (26.0)	11 (25.6)	1.00
Family history (n, %)	10 (9.6)	1 (2.3)	0.24
Sustained VT (n, %)	59 (56.7)	30 (69.8)	0.20
Comorbidities			
Hypertension (n, %)	15 (14.4)	5 (11.6)	0.85
Diabetes (n, %)	1 (1.0)	2 (4.7)	0.42
Coronary artery disease (n, %)	2 (1.9)	0 (0)	0.89
Heart failure (n, %)	10 (9.6)	6 (14.0)	0.63
Smoking (n, %)	21 (20.2)	9 (20.9)	1.00
Alcohol (n, %)	11 (10.6)	5 (11.6)	1.00
Anti-arrhythmic drugs			
Class I (n, %)	16 (15.4)	6 (14.0)	1.00
Class II (n, %)	41 (39.4)	20 (46.5)	0.54
Class III (n, %)	43 (41.3)	12 (27.9)	0.18
Class IV (n, %)	1 (1.0)	2 (4.7)	0.42
Characteristics on electrocardiography			
RBBB (n, %)	39 (37.5)	12 (27.9)	0.36
TWI-A (n, %)	67 (64.4)	26 (60.5)	0.79
TWI-I (n, %)	38 (36.5)	19 (44.2)	0.50
NSVT (n, %)	45 (43.3)	19 (44.2)	1.00
SDNN (ms)	125.5 (100.0, 145.8)	114.0 (94.0, 141.0)	0.40

Characteristics on echocardiography			
RV enlargement (n, %)	59 (56.7)	21 (48.8)	0.49
RVOT dyskinesia (n, %)	49 (47.1)	22 (51.2)	0.79
RV free wall dyskinesia (n, %)	68 (65.4)	29 (67.4)	0.96
PAH (n, %)	32 (30.8)	13 (30.2)	1.00
Tricuspid regurgitation (n, %)	70 (67.3)	28 (58.1)	0.39
Mitral regurgitation (n, %)	30 (28.8)	15 (34.9)	0.60
LVDD (mm)	47.0 (44.0, 51.0)	47.0 (44.0, 50.0)	0.67
LVEF (%)	60.9 (52.8, 65.8)	62.1 (56.0, 65.8)	0.46

sVT, sustained ventricular tachycardia; ICD, implantable cardiac defibrillator; RFCA, radiofrequency catheter ablation; RBBB, right bundle branch block; TWI-A, T wave inversion on anterior leads; TWI-I, T wave inversion on inferior leads; NSVT, non-sustained ventricular tachycardia; SDNN, Standard deviation of NN intervals; RV, right ventricle; PAH, pulmonary artery hypertension; LVDD, left ventricular end diastolic dimension; LVEF, left ventricular ejection fraction.

Supplementary table 2

Baseline characteristics of patients randomized in the model group

Variables	sVT group (n = 59, 56.7%)	Non-sVT group （n = 45, 43.3%）	P value
Age (years)	45.8±14.3	45.1±15.9	0.82
Male (n, %)	55 (93.2)	25 (55.6)	< 0.001
History (years)	4.0 (1.0, 10.0)	5.0 (1.0, 9.0)	0.88
Age of diagnosis (years)	41.0 (30.0, 48.0)	41.0 (28.0, 51.0)	1.00
Syncope (n, %)	27 (45.8)	17 (37.8)	0.54
ICD (n, %)	19 (32.2)	5 (11.1)	0.02
RFCA (n, %)	23 (39.0)	4 (8.9)	0.001
Family history (n, %)	8 (13.6)	2 (4.4)	0.22
Comorbidities			
Hypertension (n, %)	8 (13.6)	7 (15.6)	1.00
Diabetes (n, %)	0 (0)	1 (2.2)	0.89
Coronary artery disease (n, %)	2 (3.4)	0 (0)	0.60
Heart failure (n, %)	3 (5.1)	7 (15.6)	0.14
Smoking (n, %)	19 (32.2)	2 (4.4)	0.001
Alcohol (n, %)	11 (18.6)	0 (0)	0.006

Anti-arrhythmic drugs			
Class I (n, %)	11 (18.6)	5 (11.1)	0.44
Class II (n, %)	28 (47.5)	13 (28.9)	0.09
Class III (n, %)	32 (54.2)	11 (24.4)	0.004
Class IV (n, %)	1 (1.7)	0 (0)	1.00
Characteristics on electrocardiography			
RBBB (n, %)	23 (39.0)	16 (35.6)	0.88
TWI-A (n, %)	44 (74.6)	23 (51.1)	0.02
TWI-I (n, %)	25 (42.4)	13 (28.9)	0.23
NSVT (n, %)	26 (44.1)	19 (42.2)	1.00
SDNN (ms)	114.0 (88.0, 136.0)	132.0 (115.0, 151.0)	0.003
Characteristics on echocardiography			
RV enlargement (n, %)	42 (71.2)	17 (37.8)	0.001
RVOT dyskinesia (n, %)	26 (44.1)	23 (51.1)	0.61
RV free wall dyskinesia (n, %)	40 (67.8)	28 (62.2)	0.70
PAH (n, %)	23 (39.0)	9 (20.0)	0.06
Tricuspid regurgitation (n, %)	43 (72.9)	27 (60.0)	0.24
Mitral regurgitation (n, %)	15 (25.4)	15 (33.3)	0.51
LVDD (mm)	47.0 (42.0, 51.0)	48.0 (45.0, 51.0)	0.30
LVEF (%)	60.0 (52.0, 65.8)	63.0 (54.0, 65.9)	0.53

sVT, sustained ventricular tachycardia; ICD, implantable cardiac defibrillator; RFCA, radiofrequency catheter ablation; RBBB, right bundle branch block; TWI-A, T wave inversion on anterior leads; TWI-I, T wave inversion on inferior leads; NSVT, non-sustained ventricular tachycardia; SDNN, Standard deviation of NN intervals; RV, right ventricle; PAH, pulmonary artery hypertension; LVDD, left ventricular end diastolic dimension; LVEF, left ventricular ejection fraction.

Supplementary table 3

Baseline characteristics of patients randomized in the validation group

Variables	sVT group (n= 30, 69.8%)	Non-sVT group （n=13, 30.2%）	P value
Age (years)	46.2±17.4	44.7±16.2	0.79
Male (n, %)	23 (76.7)	8 (61.5)	0.52
History (years)	3.0 (1.0, 10.0)	3.0 (1.0, 8.5)	0.55
Age of diagnosis (years)	43.0 (27.3, 49.0)	37.0 (27.5, 55.0)	0.81
Syncope (n, %)	12 (40)	4 (30.8)	0.82
ICD (n, %)	12 (40)	1 (7.7)	0.08

RFCA (n, %)	8 (26.7)	3 (23.1)	1.00
Family history (n, %)	0 (0)	1 (7.7)	0.66
Comorbidities			
Hypertension (n, %)	2 (6.7)	3 (23.1)	0.31
Diabetes (n, %)	0 (0)	2 (15.4)	0.16
Coronary artery disease (n, %)	0 (0)	0 (0)	NA
Heart failure (n, %)	4 (13.3)	2 (15.4)	1.00
Smoking (n, %)	7 (23.3)	2 (15.4)	0.86
Alcohol (n, %)	3 (10.0)	2 (15.4)	1.00
Anti-arrhythmic drugs			
Class I (n, %)	4 (13.3)	2 (15.4)	1.00
Class II (n, %)	15 (50.0)	5 (38.5)	0.72
Class III (n, %)	12 (40.0)	0 (0)	0.02
Class IV (n, %)	2 (6.7)	0 (0)	0.87
Characteristics on electrocardiography			
RBBB (n, %)	10 (33.3)	2 (15.4)	0.40
TWI-A (n, %)	21 (70.0)	5 (38.5)	0.11
TWI-I (n, %)	13 (43.3)	6 (46.2)	1.00
NSVT (n, %)	13 (43.3)	6 (46.2)	1.00
SDNN (ms)	108.9 (90.5, 127.8)	139.0 (105.2, 158.0)	0.04
Characteristics on echocardiography			
RV enlargement (n, %)	19 (63.3)	2 (15.4)	0.01
RVOT dyskinesia (n, %)	14 (46.7)	8 (61.5)	0.57
RV free wall dyskinesia (n, %)	23 (76.7)	6 (46.2)	0.11
PAH (n, %)	9 (30.0)	4 (30.8)	1.00
Tricuspid regurgitation (n, %)	20 (66.7)	5 (38.5)	0.17
Mitral regurgitation (n, %)	11 (36.7)	4 (30.8)	0.98
LVDD (mm)	46.5 (39.8, 50.0)	50.0 (45.5, 53.0)	0.15
LVEF (%)	61.9 (54.5, 66.4)	62.7 (58.5, 65.8)	0.86

sVT, sustained ventricular tachycardia; ICD, implantable cardiac defibrillator; RFCA, radiofrequency catheter ablation; RBBB, right bundle branch block; TWI-A, T wave inversion on anterior leads; TWI-I, T wave inversion on inferior leads; NSVT, non-sustained ventricular tachycardia; SDNN, Standard deviation of NN intervals; RV, right ventricle; PAH, pulmonary artery hypertension; LVDD, left ventricular end diastolic dimension; LVEF, left ventricular ejection fraction.

Supplementary Figure 1

Study flow diagram

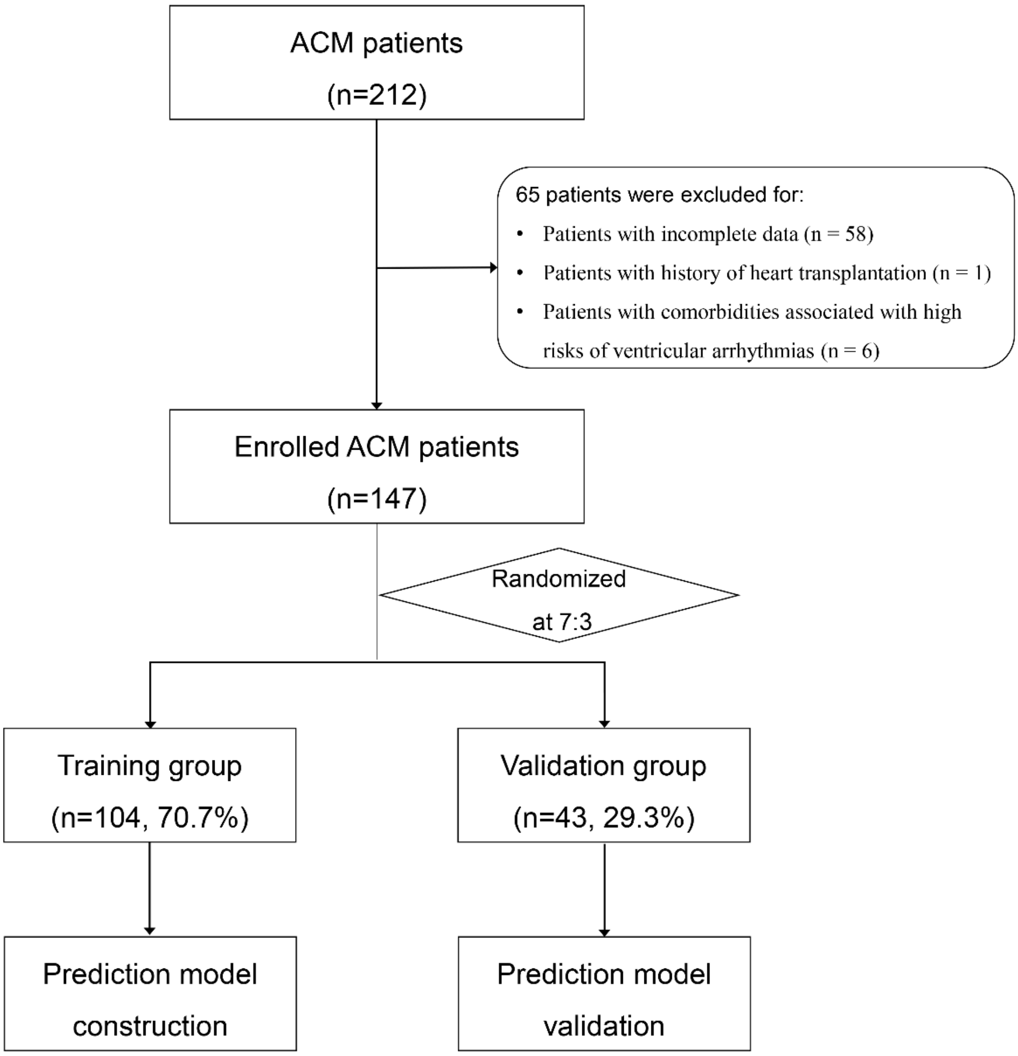

Supplement: Supplementary file 1 [file Datasheet1.pdf]
